# Emotional Availability and Play in Mother–Child Dyads with ASD: Changes during a Parental Based Intervention

**DOI:** 10.3390/brainsci10120904

**Published:** 2020-11-24

**Authors:** Silvia Perzolli, Giulio Bertamini, Simona de Falco, Paola Venuti, Arianna Bentenuto

**Affiliations:** 1 Laboratory of Observation, Department of Psychology and Cognitive Science, Diagnosis and Education (ODFLab), University of Trento, 38068 Rovereto, Italy; giulio.bertamini@unitn.it (G.B.); simona.defalco@unitn.it (S.d.F.); paola.venuti@unitn.it (P.V.); arianna.bentenuto@unitn.it (A.B.); 2 Center for Information Technology, Bruno Kessler Foundation (FBK), University of Trento, 38123 Trento, Italy

**Keywords:** Autism Spectrum Disorder (ASD), mother–child interaction, parental involvement, predictors of change

## Abstract

(1) Background: Parental involvement during intervention with children with Autism Spectrum Disorder (ASD) has been demonstrated to be fundamental for children’s developmental outcomes. However, most research focused on child gains especially considering cognitive functioning and symptoms severity, whereas parental and dyadic changes during intervention need further investigation. (2) Methods: 29 mothers in interaction with their preschool children with ASD were analyzed through two standardized behavioral and observational measures to evaluate the dyadic Emotional Availability (EA) and play skills before (T1) and after (T2) a parental-based intervention. (3) Results: Results revealed mothers increased affective quality and major awareness in understanding the signals produced by the child, that in turn was more responsive, involving also using more complex play strategies. Interestingly, the role of specific factors able to predict parental characteristics was investigated, pointing out the important contribution of mothers’ perceptions of having a difficult child and child language communicative abilities. (4) Conclusions: the study enhances knowledge about child and caregiver variables that impact on dyadic outcomes, identifying important target areas to be addressed during intervention. Further, our results suggest that a parental-based intervention supports and facilitates improvements in both children’s and caregivers’ affective quality and cognitive abilities.

## 1. Introduction

Autism Spectrum Disorder (ASD) is a neurodevelopmental disorder characterized by qualitative impairments in social interaction and communication alongside a pattern of restricted and repetitive behaviors and interests [1]. The fundamental role of caregiver–child interactions on child cognitive, social and affective development is well established considering children with Typical Development (TD) [2,3], with other Developmental Disabilities (DD) such as Down Syndrome [4,5] and with Autism Spectrum Disorder (ASD) [6,7]. However, core symptoms of ASD dramatically impact on the child’s ability to interact with significant others, especially with parents, inducing maladaptive caregiver–child interactive circuits that need to be restored in order to guarantee effective emotional exchanges [8,9]. Children with ASD tend to be socially less involving, less responsive and they have the tendency to decline, reject or ignore their caregivers’ social initiatives [9]. Furthermore, children with ASD have difficulties in sharing attention on an object with an interactive partner [10] and they often appear to be wholly focused on objects [8,11]. Consequently, interventions teaching parents strategies designed to increase time in joint engagement might be crucial [12]. Interestingly, recent at-risk studies pointed out a developmental picture revealing that children with eventual autism and their primary caregiver interact with each other in some ways that depart from a typical trajectory early in the child’s first years of life [13]. In addition to this, ASD symptomatology impacts significantly also on children’s play abilities, in fact children with ASD often engage with objects in repetitive ways and fail to develop creative and symbolic engagement with objects [14]. Also, play provides a platform for social engagement with others [15], and indeed, socially connected play and social behavior with others are particularly impaired in children with autism [8,14,16]. Taken together, these difficulties constitute a daily challenge for parents to engage children in joyful syntonic activities, influencing stress levels perceived by parents as well as the general emotional climate to which the child is exposed [17]. 

### 1.1. Affective Quality in Mother–Child Dyads with ASD

First interactions with parents have a unique and fundamental function for the overall development of children with typical development and of children with autism. Several research works investigated the role of parent–infant interaction focusing on mothers as primary caregiver. However, the recent socio-cultural changes implicate an increasing involvement of fathers in child rearing. In line with this, scientific literature highlights the relevance of fathering for child psychological development in children with and without developmental disabilities [18,19,20,21,22]. These results pointed out both similarities and differences in the interactive modalities that may support specific aspects of child development. In the context of ASD, some research highlighted that mothers tend to be more directive [23,24], displaying more intrusive and controlling behaviors and physical attempts to catch the child’s attention [4,25]. Mothers of children with ASD were also found to be as sensitive as mothers of children with typical development [26] or other developmental disabilities [27]. These findings result as particularly important, given the impact of parent scaffolding and sensitivity, for the emotional development of children with ASD [17]. However, recent studies showed that mothers in the group of children with ASD demonstrated lower sensitivity, assessed through the scheme of Karreman, highlighting their difficulties with timing and quality of play intervention [28]. This scale contains seven dimensions of maternal parenting rated on a 7-point Likert scale including warmth, sensitivity, provision of structure and limit-setting and it is applied on video-recorded observations during home-visit mother–child interactions using different play materials. This instrument was found to be particularly sensitive in capturing mothers’ characteristics, but it does not capture how children respond to their mother’s interactive strategies.

In the last few years, research focused on the dyadic nature of the mother–child relationship, in which the child clearly plays an active role [29,30]. In line with this, the construct of the Emotional Availability (EA) refers to a solid empirical basis (attachment theory, theory of emotions, maternal sensitivity) but focuses on the quality of emotional exchanges between parent and child, on their reciprocal accessibility, as well as their ability to understand and respond to each other communicative signals [31]. The construct was later operationalized by Biringen and colleagues in the Emotional Availability Scales (EAS, [32] see measures for details). Research on EA Scales has been conducted in a variety of contexts and validated in over twenty countries (Europe, Asia, subcultures of the United States etc.) [33,34,35,36] and because of this the EA Scales are one of the most widely used instruments for assessing the interaction within the parent–child dyad. However, to our knowledge only few studies have analyzed EA in mother–child with ASD dyads [21,27,37,38,39] and no studies investigated EA in a longitudinal design in these dyads. Furthermore, EA is found to be predictive of various positive developmental outcomes such as attachment security [39,40], emotion expressions and regulation [41] and school readiness [40]. EA parental levels seem also to modulate the functionality of neural circuits involved in executive functioning, especially in response inhibition [42]. These results suggest that intervention aiming to increase maternal EA should begin early in the child’s life [43].

### 1.2. Cognitive Elements in Mother–Child Dyads with ASD: The Role of Play

Another important context for the child’s early social and cognitive development is play. It provides a motivating opportunity for child learning and advanced levels of play are associated with greater cognitive and language development [16]. At first, children are generally more focused on physical properties of toys and their exploration, but later they engage more in symbolic activities that need higher representational abilities and cognitive skills. Despite deviations in play behaviors in children with ASD, more skills in symbolic play are reported when play is scaffolded by an adult [44] and more systemic instructions are required to support the play with peers [45]. Recent findings revealed also that mothers of children with ASD adapted their activities to their child’s sophistication level [46]. These mothers seem to use fewer symbolic solicitations than mothers of children with typical development but a positive correlation between maternal verbal solicitations of symbolic play and children’s actual symbolic play was found only in children with ASD, highlighting a fundamental role of the caregivers in cognitive aspects in dyads with children with ASD. Considering this, [47] operationalized a scale for play behavior that follows the progression from simple manipulation of toys, to recognition of conceptual relationships between objects (i.e., functional play and combinatory play), to increasingly decontextualized play (i.e., symbolic or pretend play). This code allows individual scoring of caregiver and child levels of play to see how parents adapt their level to child functioning, and also to investigate how the child responds to a caregiver’s behavioral characteristics.

In general, mother–child interactions may be influenced by the fact that mothers of children with ASD tend to have higher levels of stress compared to mothers of children with typical development [28,48]. Consequently, stress levels may lead parents to feel more negative emotions and to be more irritable and upset [49], directly influencing child behavioral problems [28,50]. In line with this, maternal stress and negative feelings over the child were negatively associated with the emotional availability and particularly on structuring skills using verbal and non-verbal techniques [38]. All together these findings strengthen the importance of finding shared strategies for parents in dealing with their children, given their positive impact on stress reduction [51].

### 1.3. Parental Involvement during Intervention with Children with ASD

Given the influence of parent’s qualities and dyadic characteristics on child developmental outcomes, recent findings strengthened the importance of involving caregivers during the intervention in order to increase dyadic levels of syntonization and to extend the acquisition of competencies also in naturalistic contexts (e.g., home) [52,53,54]. Further, parental involvement during intervention seemed to be extremely important in order to guarantee the adaptation to child’s difficulties and impairments, allowing the child to respond with enhanced communicative and social development [55], long-term symptom reduction [56], and to generalize these outcomes across settings [57]. Further, recent literature documented that marked difficulties in social communication and responsiveness in parents of children with ASD [58] might create a potential barrier to care for their children. In line with this, recent findings suggested that an enhanced version of parental involvement was able to guarantee more prominent results considering caregivers. Interestingly, the authors found a significant relationship between the degree of change in parental interaction and the rate of child’s improvement [59], underlying the importance of the dyadic relational aspects in child developmental outcomes. Moreover, some research showed that without involving caregivers these variables tend to remain more stable over time [55,56,57]. Because parents are so important in ensuring success and good prognosis, it is critical to include them throughout the intervention process. As a matter of fact, these findings shed new light on the idea that if parents are adequately informed during intervention and if they constantly deliver intervention strategies in naturalistic contexts, the intensity of the intervention dwindles on intervention outcomes. Caregivers, in fact, may carry on teaching competencies to their children in the home context, improving parent–child interactions and actually increasing the amount of treatment they receive. Recently, Naturalistic Developmental Behavioral Interventions (NDBI) are underlying the role of interactive aspects. Different works on efficacy showed significant gains in developmental outcomes and in symptoms severity [60,61,62,63,64,65]. However, in general the evaluation of treatment response is mainly focused on child outcomes demonstrating the improvements in both child’ social engagement and their cognitive development [66], often without deepening dyadic and caregivers’ variables associated with the response. Child gains are, in fact, predominantly assessed through developmental outcome measures using standardized instruments such as Autism Diagnostic Observation Schedule (ADOS-2, [67]) and Griffiths Scales (GMDS-ER, [68]). However, these instruments in the assessment of treatment’s outcomes might suffer from the issue of sensitivity [69]. Standardized diagnostic and cognitive test items are, in fact, neither proximal to the treatment nor necessarily sensitive to small changes in social communication and interactive skills that may be occurring as treatment progresses. For this, the detection of change may be enhanced by using observational measures of social responsiveness [70]. In line with this, the analysis of the dyad’s interactive component through observational and behavioral measures might represent a very sensitive-to-change instrument to assess caregiver and child improvements in the relational context.

To conclude, at this point it is also important to identify caregivers’ characteristics that impact on intervention outcomes given the paucity of empirical research. For example, some research pointed out that higher parental age may be linked to more successful interactions and this could be due to their greater experience in parenting [71]. Further, higher educational level [71] and lower stress levels in parents [72] seemed to be associated with greater developmental outcomes. Also, different caregiving styles, such as higher levels of parental sensitivity, were also found to be related with better child communications abilities [27,29] and joint engagement [73]. Further, the increase of maternal age is associated with the increase of structuring abilities in interaction with the child with typical development [74,75,76]. In fact, as pointed out in three longitudinal studies that monitor mothers’ interactive skills at different time points, it seems that the increase of maternal age may be associated with increased structuring abilities. Moreover, mothers’ stress and depressive symptoms predicted the warmth and criticism toward their child, and the general well-being of the primary caregiver seemed to restrict or promote the involvement of the other caregiver in the ASD context [77]. More empirical evidence and more exploration is needed considering caregivers characteristics and dyadic factors.

### 1.4. Aims and Hypotheses

In accordance with the above, the aim of the present work is to assess an Early Intensive Intervention with Parent Involvement focusing on caregivers and dyadic outcomes through two observational standardized instrument that allow the evaluation of the affective quality (measured through the Emotional Availability Scales, EAS, [32]) within the dyad and the play skills abilities (assessed through the play code, [47]). First of all, in the present study we want to examine how the implementation of a parental-based intervention, that provides active participation of the caregiver into the therapeutic setting, impacts on interactive characteristics within the dyad and how this relationship evolves over time, given that the majority of empirical work is conducted on child outcomes only. Then, we want to examine the impact of child and caregiver factors on the emotional availability and on the play skills abilities given the persistent need to examine in details some factors that might predict, moderate and mediate intervention effectiveness for children and their parents. On this basis, we hypothesize as follows. First of all, we want to investigate if mothers and their children will improve specific interactive modalities during intervention.

1. In line with previous findings that include caregivers into the therapeutic setting [55,56,57], and in line with the theoretical framework of the implemented intervention that focuses on the syntonization between adult and child’s needs, we expect that mothers will increase their awareness of timing during the interaction with the child, catching child’s signals and respecting his/her time given the possibility for parents to experiment themselves in functional interaction with their children.
2. Further, considering that during intervention therapists provide caregivers appropriate hints and suggestions to interact and play with their children in a functional way, we also predict that mothers will increase their general levels of Emotional Availability, especially considering structuring and non-intrusiveness scales.
3. With respect to the child, in line with previous findings that depicted positive change in child socio-communicative behaviors [78,79,80,81] we expect to find improvements in the child’s level of responsiveness and in the use of different communicative strategies (e.g., eye-contact looking, body positioning, verbal involvement) to involve the caregiver during an interactive exchange.

Second, we want to examine the evolution of child cognitive play abilities during intervention.

1. In particular, we expect that during the intervention children will increase their level of symbolic play, consequently decreasing their level of exploratory play during the interaction with the caregiver, given that the intervention focuses also on a specific work on cognitive abilities necessary to be able to play with more advanced skills.
2. We further expect that mother and child scores will be more related after intervention, indicating an increased adaptation by the adult to the child’s level of play.

Finally, we want to investigate the impact of child and caregivers’ factors on the affective quality during their interaction measured through the Emotional Availability Scales. In particular,

1. Considering the child, we expect that the communicative aspects of the child in interaction with the caregiver may impact on the child’s scales of Emotional Availability, given the impact of verbal and non-verbal communicative aspects on dyadic functional relationships.
2. Further, on the basis of previous literature that reported that higher levels of parental stress may impact on adult interactive modalities considering children with typical development [82] and children with ASD [83], we want to investigate the parental stress with respect to the adult Emotional Availability, and the specific stress dimensions that may impact on parent’s modalities during the interplay with the child.

## 2. Materials and Methods

### 2.1. Participants

This study involved 29 Italian preschool children (27 males and 2 females) with Autism Spectrum Disorder (ASD) (M chronological age = 46.65 months, SD = 11.1; M mental age = 33.76 months, SD = 9.15) and their mothers, exposed to an early intensive treatment with parent involvement (M chronological age = 38.31 years, SD = 4.89) (see Table 1). All participants were recruited at the Laboratory of Observation, Diagnosis and Education (ODFLab), a clinical and research center of the Department of Psychology and Cognitive Science (University of Trento, Italy) specialized in functional diagnosis of neurodevelopmental disorders, in particular ASD, where families voluntarily go to in order to receive an assessment of their child functional profile. Moreover, the laboratory currently implements a parental based early intensive intervention in line with Naturalistic Developmental Behavioral Interventions (NDBI) [66].

The diagnosis of ASD of the children in the present work was confirmed through clinical judgment by an independent clinician based on the DSM-5 criteria for Autism Spectrum Disorder, as well as through the administration of the Autism Diagnostic Observation Schedule (ADOS-2; [67]). Recruitment of participants was done on a voluntary basis through advertisements in the waiting room of the Laboratory. Then, a dedicated meeting will be scheduled with a referent not involved in the clinical process in order to explain the objectives of the research. Finally, participants had to sign a written consent.

### 2.2. Procedure

All procedures of this study were in accordance with the ethical standards of the Italian Association of Psychology (AIP), with the ethical standards of the Ethics Committee of the University of Trento (Italy) and the last version of the Declaration of Helsinki [84]. In order to determine children’s developmental level, the Griffith Mental Development Scale-Edition Revised [68] was administered to all children. Further, Autism Diagnostic Observation Schedule (ADOS-2; [67]) was administered in order to certify the presence of Autism Spectrum Disorder and to specify the severity level. On the basis of the level of language development and the chronological age of children, ADOS Toddler, Module 1 and Module 2, were applied. Further, in order to assess the affective quality within the dyad the Emotional Availability Scales (EAS, [32]; see measures for details) were applied to ten minutes of video-recorded interactions between the child and the caregiver in which mothers were asked to spontaneously play with their child with a standard set of toys as if they were at home. In the same interaction sequence, the Play code ([47] see measures for details) was also applied in order to assess the levels of both child and caregiver’s exploratory and symbolic play. The coding is randomly assigned to two independent observers that codify the interactions after receiving a specific training considering both the application of the Emotional Availability Scales (EAS) and the Play Code, and after having reached a significant level of interrater reliability. Two-way mixed effects ICC with absolute agreement has been used [85]. Two independent observers codified four target videos with a total duration of about 120 min as indicated in the EAS training [32]. The coefficient ranged from 0.84 to 0.92.

Considering play the average kappa’s between coders for the play levels ranged from 0.79 to 0.88 for training videos. The above-mentioned measures are applied before intervention (T1), during the first diagnostic and functional assessment and after an average of 11.03 (SD = 3.00) months of intervention (T2) to both children and caregivers, in order to investigate the evolution of dyadic factors during intervention and the impact of affective characteristics on cognitive play skills.

### 2.3. ODFLab Parental Based Intensive Intervention

ODFLab implements an “Italian Model of Intervention” which combines empirically validated scientific principles together with guidelines in accordance with the Italian sanitary system [52,86,87]. This intervention integrates behavioral, developmental and relationship-based principles, according to the key elements of the American Early Start Denver Model [81,88]. Further, in order to restore caregiver–child maladaptive interactive circuits and to strengthen the generalization of child competencies, the intervention involves caregivers into the therapeutic setting together with the child. The ground idea is that if caregivers have the possibility to learn adequate strategies to deal with their children, they may effectively make use of them in more naturalistic contexts (e.g., home). With this in mind, the intervention includes specific activities for the child (speech therapy, music therapy, cognitive activities and emotional and social play for 4–6 h per week) and parent involvement into the therapy room (at least 2 h per week). Differently from parent-mediated intervention and parent training, the parental involvement does not require home assignments nor fidelity schedules and the intervention is entirely delivered by the therapist. In fact, during these weekly sessions the therapist remains the key figure that structures activities but he/she also creates opportunities for the caregivers and the child to interact and play together. Consequently, the caregivers have the possibility to experience more functional interactions with the child characterized by more adequate proposals and major awareness of the child’s individual timing. In turn, such changes may increase dyadic shared pleasure, parents’ self-efficacy and motivation to interact with their child, while reducing stress and frustration levels.

Importantly, these interactions between adult and child are discussed together with a psychotherapist every two weeks through the video-feedback procedure. Thanks to this, the intervention works on functional dyadic characteristics and consequently on parental representation of both the child and the caregivers in their role. In fact, during these meetings the parents have the possibility to analyze specific child’s avoidant, non-respondent or other problematic behaviors, understanding child’s signals together with the therapist. Therefore, they have the possibility to build a more truthful image of the child and of himself/herself as a parent. In addition to this, meetings with parents of all children are organized in order to create a moment of sharing feelings, emotions, experiences, fears and uncertainties and experiencing the sensation of not being alone in dealing with children with ASD. The intervention is delivered by trained psychologists after receiving specific licenses on developmental models of intervention for children with ASD. Finally, the team is constantly supervised at least once every month by an expert psychotherapist.

### 2.4. Measures

#### 2.4.1. Griffiths Mental Development Scales

Child cognitive development before and after intervention is assessed through the Griffiths Mental Development Scales (GMDS-ER; [68], see Table 2). The GMDS-ER are developmental scales normalized also in an Italian sample and are administered to the child in a laboratory setting through semi-structured activities designed to evaluate different aspects of mental development in infants and children. They provide Z-scores relative to 6 subscales in the main developmental areas: Locomotion; Personal–Social; Communication and Listening; Eye–Hand Coordination; Performance; and Practical Reasoning. These scales provide a Global Quotient and a developmental age-equivalent—allowing to detect developmental delays—as well as specific quotients and developmental age-equivalents for each of the 6 subscales. All scores are standardized (M = 100; SD = 15).

#### 2.4.2. Autism Diagnostic Observation Schedule-2

Child severity of symptoms is assessed before and after intervention through the Autism Diagnostic Observation Schedule (ADOS-2; [67], see Table 2), golden standard instrument for the diagnosis of ASD. The administration of this tool is carried out by trained psychologists after an official ADOS course. The instrument provides different modules according to child chronological age and expressive level of language. Each module gives a total score used for the ADOS diagnostic classification (Autism–Autism Spectrum–Non Spectrum). This score is transformed in the comparison score in order to perform comparisons among different modules and classify the severity of symptoms in three categories (mild, moderate or severe).

#### 2.4.3. Emotional Availability Scales

Mother–child Emotional Availability (EA) is assessed before and after the early intensive intervention with parent involvement through the Emotional Availability Scales (EAS; [32]). EA is a relational construct that refers to the quality of emotional exchanges between parent and child. It focuses on their reciprocal accessibility and their ability to understand and respond appropriately to each other’s communicative signals (31). The EA Scales are created to observationally measure and operationalize the construct of Emotional Availability and consist of four scales for adults that include sensitivity, structuring, non-intrusiveness and non-hostility, as well as two scales for children, responsiveness and involvement, with 7 subscales that measures specific dimensions of the main scale (see Table 3). The main scales are scored from 1 (lowest EA) to 7 (highest EA) on a Likert scale; the first two subscales are scored from 1 to 7, however the other 5 subscales are scored from 1 (lowest EA) to 3 (highest EA). Midpoints were also used in the present study as they are highly recommended especially for children with disabilities [89,90]. Adult Sensitivity refers to the ability to capture and respond adequately to the child’s communicative signals and to the caregiver’s ability to be emotionally connected to the child [91]. Adult Structuring refers to the parent’s ability to promote and organize the child’s activities by furnishing appropriate prompts and suggestions during interaction, without limiting the child’s autonomy. Non-intrusiveness refers to the ability of the parent to be aware of the best time to fit in the interaction without being too demanding nor directive. Finally, Non-hostility refers to the ability to interact with the child without showing over nor covert signs of hostility. The child’s scales comprise Responsiveness and Involvement. Responsiveness refers to the child’s manifestation of clear signs of pleasure during the interaction with the caregiver and measures how often the child responds to parents’ suggestions. Finally, Involvement refers to the child’s ability to actively engage and involve the parent into interaction through different modalities (eye-contact looking, verbal involvement, body positioning). In general, optimal levels of EA (7) are characterized by the presence of an ideal quality of affect and support, as well as child’s optimal levels of responsiveness and involvement. Moderate levels (5–6) refers to good modalities of parents in interaction with the child that shows appropriate but not ideal strategies for involving caregivers. Apparent/inconsistent levels of EA (4) are characterized by the adult’s inconsistency in their ability to lead the child, that, in turn, shows both positive and negative approaches to draw the adult’s attention. Low levels (3/2.5) or very low levels (2–1) of EA are typical of caregivers that show insensitive and unavailable affect and children are characterized by worry, anxiety and distress in their responsiveness and their involving strategies might be inappropriate or nonexistent.

### 2.5. Play

Mother–child Play skills are assessed before and after intervention through the Play code [47], an operationalized scale that follows the sequence of play from simple exploration of toys, to identification of relationships between objects (i.e., functional play and combinatory play), to increasingly decontextualized play (i.e., symbolic or pretend play). The code allows to score caregiver and child levels of play individually, but also to investigate whether parents appropriately adapt to the child’s intentionality and level of functioning, as well as how he/she responds to parent’s interactive proposals. The play levels considered in this code are described briefly as follows:

Level 1. Unitary functional activity: considers effects that are unique to a single object (e.g., dialing a telephone).

Level 2. Inappropriate combinatorial activity: refers to the inappropriate juxtaposition of two or more objects (e.g., putting the cup on the telephone).

Level 3. Appropriate combinatorial activity: concerns the appropriate juxtaposition of two or more objects (e.g., putting the handset on the telephone).

Level 4. Transitional play: refers to the approximation of pretend play but the action is not concluded (e.g., putting the telephone handset to ear without vocalization).

Level 5. Self-directed pretense: relates to the pretense activity that is directed toward self (e.g., drinking from an empty cup).

Level 6. Other-directed pretense: pertains to the pretense activity that is now directed towards someone or something else (e.g., putting a doll to sleep).

Level 7. Sequential pretense: concerns child’s ability to connect two or more pretense actions (e.g., pouring into an empty cup from the teapot and then drinking).

Level 8. Substitution pretense: refers to the child’s ability to include one or more object substitutions (e.g., pretending a cup is a telephone and talking into it).

Level 9. When no action is taken within the dyad (e.g., child is running around the room in an afinalistic way and no object is touched).

Play levels from 1 to 4 are considered an index of the child’s exploratory play, whereas levels from 5 to 8 reflect the quantity of symbolic play displayed during the interaction. The trained observers used BORIS (Behavioral Observation Research Interactive Software, [92], an open-source event logging software for video/audio coding and live observations. It allows to code continuously by noting play level as well as start and end times (accurate to 1 s). Minimum play time is fixed at 1 s, after that a specific level of play is coded until there is a break longer than 10 s during which neither the child nor the caregiver touches an object. For each level of play four measures are extracted: the absolute frequency, the proportional frequency, the absolute duration, and the proportional duration. These scores are later normalized through the proportion of maximum scaling (“POMS”), a min-max normalization method [93,94] that transforms each index to a metric from 0 to 1 through the following formula: POMS = [(observed − minimum)/(maximum − minimum)]. This method is therefore useful to have a monotonic metric and to avoid problems concerning “classical” standardization in longitudinal design [89]. A general index for each play level is then computed averaging the standardized scores of the four measures (i.e., absolute frequencies, proportional frequencies, absolute durations and proportional durations). POMS indexes are then grouped in two main indexes: exploratory play, computed as the average of levels from 1 to 4, and symbolic play, computed as the average of levels from 5 to 8. These indexes represent quantitative measures of both the amount and duration of the different types of play, accounting both for absolute and proportional measures of the interplay.

### 2.6. Parental Stress Index-Short Form

The Parental Stress Index-Short Form (PSI/SF, [95]) is a standardized instrument widely used in the clinical context for the early identification of stress in the parent–child relationship. It contains 36 items on a five-point Likert scale and it yields a Total Stress score from three scales: Parental Distress, Parent–Child Dysfunctional Interaction, and Difficult Child. In particular, the Parental Distress (PD) defines stress levels of the caregiver in his/her role as a parent but considering only personal factors, independently from the child. Parent–Child Dysfunctional Interaction (P–CDI) analyses the interaction with the child perceived by the parent as difficult and problematic (e.g., when parents consider themselves rejected by the child). Difficult Child (DC) examines some characteristics of the child’s behaviors and the perception the parent has to have a child with a difficult temperament.

### 2.7. Statistical Analysis

Data has been checked for normality (i.e., Shapiro test) and homogeneity of variances (i.e., Levene test). When assumptions for parametric tests were met, Welch T test for dependent samples has been used for longitudinal analysis, as it is recommended in psychological research with relatively small samples to reduce the probability of Type I Error [96]. Otherwise, paired Wilcoxon signed rank with continuity correction has been used to verify differences between T1 and T2. Effect sizes have been calculated using R-squared.

A Bayes Factor analysis has been performed to investigate evidence supporting the null vs. alternative hypothesis given the observed data as a way to improve inferential statistics.

Data has been analyzed using R [97]. The Bayes Factor (BF) analysis has been performed using the package ‘Bayes Factor’ and will be interpreted following the proposal of Harold Jeffreys revised by Lee and Wagenmakers [98].

Linear mixed-models have been fitted to investigate longitudinal relationships between child’s play abilities on developmental outcomes and, in turn, child’s cognitive skills on caregiver Emotional Availability Scales together with parental stress levels. Models have been fitted using the ‘lme4’ package, with the ‘lmerTest’ and the ‘car’ packages to compute *p*-values, Bayesian Information Criteria (BIC), marginal R-squared and the ANOVA with Wald F test to check for model significance. Futher, the models have been checked for significance against baseline (χ^2^).

Linear correlation analysis has been performed using Pearson product-moment correlation coefficient and tested for significance.

## 3. Results

### 3.1. Longitudinal Changes: Emotional Availability Scales

#### 3.1.1. Adult’s Scales

Wilcoxon signed rank test with continuity correction revealed a significant (V(28) = 13; *p* = 0.002; *r*^2^ = 0.596; BF = 25.097) change in adult’s Sensitivity scale during intervention, indicating a medium effect. Bayes Factor (BF) analysis suggests strong evidence for the alternative hypothesis. Considering Sensitivity subscales, results revealed that “the awareness of timing” showed significant increase (V(28) = 0; *p* = 0.026; *r*^2^ = 0.454; BF = 2.952). Further, the subscale “amount of interaction” revealed a significant (V(28) = 0; *p* < 0.001; *r*^2^ = 0.669; BF > 100) change before and after the intervention, indicating a strong effect. BF analysis suggests extreme evidence for the alternative hypothesis. Considering adult Structuring scale, results exhibit a significant (V(28) = 0; *p* < 0.0001; *r*^2^ = 0.892; BF > 100) change between T1 and T2, indicating a strong effect and extreme evidence for the alternative hypothesis through the BF analysis. The investigation of the subscales revealed that the “appropriate guidance and suggestions” increased significantly (V(28) = 24; *p* < 0.0001; *r*^2^ = 0.795; BF > 100) during time, indicating a strong effect and extreme evidence for the alternative hypothesis. Also, the rate of “success of attempts” emerged to be significant (V(28) = 16; *p* = 0.003; *r*^2^ = 0.559; BF = 23.428) and the “amount of structure” also increased significantly (V(28) = 0; *p* < 0.001; *r*^2^ = 0.641; BF > 100) during play with the child, indicating a strong effect and suggesting extreme evidence for the alternative hypothesis. The subscales “remaining firm in the face of pressure” and “verbal vs. non-verbal structuring” are both significant, respectively (V(28) = 5; *p* = 0.023; *r*^2^ = 0.433; BF = 2.952) (V(28) = 0; *p* = 0.010; *r*^2^ = 0.523; BF = 2.108). With respect to the adult Non-Intrusiveness scale, results showed a significant (V(28) = 0; *p* < 0.0001; *r*^2^ = 0.886; BF > 100) change during intervention, indicating a strong effect and supporting extreme evidence for the alternative hypothesis. The subscales, and particularly results considering the ability of “following child’s lead” showed significant (V(28) = 38.5; *p* = 0.019; *r*^2^ = 0.504; BF = 5.069) increase during intervention, together with the ability to “not interrupt the child into interaction” (V(28) = 31; *p* = 0.014; *r*^2^ = 0.549; BF = 7.699) and the reduction of “physical vs. verbal interferences” (V(28) = 0; *p* = 0.019; *r*^2^ = 0.454; BF = 4.031). Finally, also the subscale “the adult is made to feel or seem intrusive” showed a significant change during intervention, revealing that the adult is less intrusive in the interplay (V(28) = 0; *p* = 0.003; *r*^2^ = 0.586; BF = 30.755), indicating a strong effect and a very strong evidence supporting the alternative hypothesis. Adult Non-Hostility scale revealed no differences in the main domain nor in the subscales (see Table 4).

#### 3.1.2. Child’s Scales

With regards to child Responsiveness scale, results showed a significant (V(28) = 21.5; *p* < 0.001; *r*^2^ = 0.716; BF > 100) improvement during intervention, indicating a strong effect. BF supports extreme evidence for the alternative hypothesis. The analysis of the subscales pointed out that “the emotional regulation of affect and behavior” resulted to be significantly (V(28) = 1; *p* = 0.022; *r*^2^ = 0.432; BF = 2.880) greater during the intervention, together with the increased “positive physical positioning” of the child towards adult (V(28) = 11; *p* = 0.036; *r*^2^ = 0.479; BF = 1.836). Moreover, the subscale that considers the degree of “quantitative responsiveness” of the child results to be highly significant (V(28) = 19; *p* = 0.001; *r*^2^ = 0.675; BF = 21.431) indicating a strong effect and strong evidence supporting the alternative hypothesis through the BF analysis. Moreover, the degree of “task orientation and concentration” without excluding the adult during the interaction is significant (V(28) = 7; *p* = 0.003; *r*^2^ = 0.566; BF = 30. 755) indicating a strong effect, with the BF analysis indicating very strong evidence for the alternative hypothesis.

To conclude, considering the child Involvement scale, the analysis revealed a significant increase (V (28) = 0; *p* = 0.002; *r*^2^ = 0.613; BF = 24.648), indicating a strong effect and a BF supporting strong evidence for the alternative hypothesis. With respect to the different subscales, results pointed out that the “simple initiative” toward the caregiver is significantly increased (V(28) = 0; *p* = 0.003; *r*^2^ = 0.557; BF = 25.097) indicating strong effect and a BF supporting strong evidence for the alternative hypothesis. Further, the subscale of the “elaborative initiative” is significantly changed as well (V(28) = 2; *p* = 0.036; *r*^2^ = 0.425; BF = 2.313). The subscale that considers the “use of adults” results significant (V(28) = 0; *p* = 0.010; *r*^2^ = 0.491; BF = 7.206) and BF analysis indicates moderate evidence for the alternative hypothesis. This indicates an increase in the degree to which the child goes to the adult for both the emotional and playful exchange. Finally, results revealed that during intervention children seemed to increase also their involving strategies to interact with the caregiver through different channels of communication such as “eye-contact looking” (V(28) = 4.5; *p* = 0.024; *r*^2^ = 0.435; BF = 2.776), “body positioning” (V(28) = 5; *p* = 0.009; *r*^2^ = 0.471; BF = 4.598) and “verbal involvement” (V(28) = 0; *p* < 0.0001; *r*^2^ = 0.742; BF > 100), the latter one indicating a strong effect. BF analysis indicates extreme evidence supporting the alternative hypothesis with respect to “verbal involvement” (see Table 5).

### 3.2. Longitudinal Changes: Play Skills

Paired sample T-tests revealed a significant decrease (t(27) = 2.922; *p* = 0.007; *r*^2^ = 0.234; BF = 6.319) in the index of child exploratory play that includes the first four levels of the Play Code during intervention. Further, the Wilcoxon signed rank test with continuity correction revealed that the index of child symbolic play, that contains more advanced levels of play skills, resulted to be significantly increased (V(27) = 79; *p* = 0.0255; *r*^2^ = 0.392; BF = 3.449) during intervention. Interestingly, we found out that the amount of exploratory play exhibited by caregivers and child is not correlated at T1 (*r* = 0.063; t(27) = −0.326; *p* = ns) but the correlation between adult and child indexes is significant at T2 (*r* = 0.591; t(27) = 3.022; *p* = 0.005) (see Table 6). Finally, we found out a moderate correlation (*r* = 0.424; t(27) = 2.436; *p* = 0.022) between the amount of symbolic play displayed by the caregiver and by the child at T1, but the correlation resulted to be particularly increased after intervention (*r* = 0.669; t(27) = 4.673; *p* < 0.001) falling into the strong range. The level of non-play, that refers to level 9 of the code, resulted to be non-significant both before and after intervention.

### 3.3. Linear Mixed Models

#### 3.3.1. Parents’ Structuring Ability is Linked to Chronological Age and Their Perception of Having a Difficult Child

The linear mixed-effect model (Model 1, see Table 7) has been fitted considering the Structuring Total Score of the Emotional Availability Scales (EAS) as dependent variable, with time, the Difficult Child Score of the Parent Stress Index (PSI) and the caregiver chronological age as fixed effects and a random effect of participants to account for repeated measures.

The model showed a significant intercept (b = 1.900; t(25.474) = 2.340; *p* = 0.027). All the predictors were significant: time (b = 0.718; t(12.196) = 9.954; *p* < 0.0001); the Difficult Child Score of the PSI (b = 0.016; t(24.079) = 2.255; *p* = 0.034) and the chronological age of the caregiver (b = 0.050; t(21.529) = 2.510; *p* = 0.020).

Anova with Wald F test confirmed significance of the intercept (F(27.315) = 5.344; *p* = 0.029) and all the predictors: time (F(14.543) = 96.451; *p* < 0.0001); the Difficult Child Score (F(26.096) = 4.5313; *p* = 0.0429) and the chronological age of the caregiver (F(23.798) = 6.226; *p* = 0.020).

The model has been compared against a baseline model (BIC = 57.402) considering only the time, and resulted to be significant (χ^2^ (6) = 9.364; *p =* 0.009; BIC = 55.365) showing an increase in the goodness of fit, with a marginal R-squared of 0.485.

#### 3.3.2. Parents’ Non-Intrusiveness is Linked to Child’s Language Ability

Model 2 (see Table 6) has been fitted considering the Non-intrusiveness Total Score of the Emotional Availability Scales (EAS) as dependent variable, with time and the child’s Language Quotient of the Griffiths Mental Development Scale (GMDS) as fixed effects, and a random effect of participants to account for repeated measures.

The model showed a significant intercept (b = 4.090; t(46.979) =2 1.277; *p* < 0.0001). All the predictors are significant: time (b = 0.771; t(26.111) = 7.045; *p* < 0.0001) and the child’s Language Quotient (b = 0.008; t(50.000) = 2.677; *p* = 0.010).

Anova with Wald F test confirmed significance of the intercept (F (47.394) = 429.756; *p* < 0.0001), time (F(28.052) = 48.834; *p* < 0.0001) and the Language Quotient (F(50.000) = 6.610; *p* = 0.013).

The model has been compared against a baseline model (BIC = 123.140) considering only the time, and resulted to be significant (χ^2^ (5) = 26.709; *p* < 0.0001; BIC = 101.400) showing an increase in the goodness of fit, with a marginal R-squared of 0.400.

#### 3.3.3. Child’s Symbolic Play Level is Predictive of Child’s Language Ability

Model 3 (see Table 6) has been fitted considering the child’s Language Quotient as dependent variable, with time and the child’s symbolic play index as fixed effects, and a random effect of participants to account for repeated measures.

The model showed a significant intercept (b = 47.218; t(41.660) = 8.046; *p* < 0.0001). Time resulted to be non-significant (b = 8.186; t(29.848) = 1.526; *p* = 0.138). The symbolic play index was significant (b = 104.376; t(46.240) = 2.810; *p* = 0.008).

Anova with Wald F test confirmed the significance of the intercept (F(41.157) = 63.895; *p* < 0.0001) and the symbolic play index (F(45.987) = 7.321; *p* = 0.010).

The model has been compared against a baseline model (BIC = 516.400) considering only the intercept, and resulted to be significant (χ^2^ (5) = 16.218; *p* = 0.0003; BIC = 508.12) showing an increase in the goodness of fit, with a marginal R-squared of 0.164.

#### 3.3.4. Child’s Responsivity is Linked to Child’s Language Ability

Model 4 (see Table 6) has been fitted considering the Responsivity Total Score of the Emotional Availability Scales (EAS) as dependent variable, with time and the child’s Language Quotient of the Griffiths Mental Development Scale (GMDS) as fixed effects and a random effect of participants to account for repeated measures.

The model showed a significant intercept (b = 2.947; t(46.702) = 13.129; *p* < 0.0001). All the predictors were significant: time (b = 0.322; t(25.642) = 2.504; *p* = 0.019) and the child’s Language Quotient (b = 0.011; t(49.983) = 3.389; *p* = 0.001).

Anova with Wald F test confirmed significance of the intercept (F(47.260) = 163.592; *p* < 0.0001), time (F(28.093) = 6.171; *p* = 0.019) and the Language Quotient (F(49.986) = 10.590; *p* = 0.002).

The model has been compared against a baseline model (BIC = 124.140) considering only the time, and resulted to be significant (χ^2^ (5) = 10.086; *p* = 0.001; BIC = 118.020) showing an increase in the goodness of fit, with a marginal R-squared of 0.260.

## 4. Discussion

Given the importance of parental involvement into the therapeutic setting [53,55,56,57,79,99,100] and the paucity of studies highlighting parental and dyadic changes during intervention, the main purpose of the present study was to investigate mother–child dyads with ASD through two standardized behavioral instruments that allow to assess both the affective quality and play skills abilities of these dyads.

With respect to our first aim, we wanted to analyze the dyadic aspects of mother–child interaction and the bidirectional influence during the exchange. These elements were assessed through the Emotional Availability Scales (EAS) before and after intervention and revealed a significant improvement in the mother’s general level of Sensitivity, especially in the subscale of awareness of timing during the interaction, waiting for the appropriate moment to propose or interrupt the child. In line with this, other studies, using different instruments than the EAS in the evaluation of treatment, reported major parent’s acceptance of the child and positive dyadic pattern [53,99]. Further, our results are also in line with findings suggesting that parents seem to show changes in their interactive strategies pre and post intervention [78,79]. In fact, we found a significant increase in mothers’ general Structuring abilities from inconsistent to moderate–good levels. Interestingly, the analysis of the subscales revealed that the improvement concerned an enhanced quality of proactive guidance and varied suggestions that lead the child in an appropriate way. Also, parents seem to adequately structure just the right amount according to the child’s need, using both verbal but also non-verbal strategies. This result could be even more important in the context of ASD. In fact, communicative abilities are often impaired, and thus verbal indications are generally not enough to effectively scaffold an appropriate interaction, making the use of nonverbal strategies play a crucial role. Consequently, interaction attempts are more appropriate and, therefore, more successful during the interaction with the child. Because EA is a dyadic construct which describes both the sending and receiving emotional signs, the structure is considered adequate when parent’s attempts are successful, in the sense that these are appreciated and welcomed by the child.

While Structuring is about guidance and mentoring, Non-Intrusiveness is about the actual over-direction, over stimulation and interference into child’s behaviors and, in the literature, these scales resulted to be particularly correlated one to the other [101]. Our results suggested that non-intrusive behaviors increased from “inconsistent” to “moderate–good” over time. In particular, mothers seemed to be more able to follow the child’s needs without interfering too much with his/her activity. In fact, fewer interruptions were observed, in favor of waiting for the perfect timing to interrupt or to propose another activity to the child. Further, the verbal and or physical interferences decreased significantly, indicating more appropriateness in the way of dealing with the child, that in turn showed fewer signs indicating that the adult is intrusive in his/her activity. This result appears to be particularly relevant considering that literature about parenting in the context of neurodevelopmental disorders consistently underlined the tendency of caregivers to be more intrusive during the interpersonal interchange [4,25]. In this framework, a significant improvement of this parental behavior, together with better scaffolding strategies and skills, represent two important elements that may actually impact on the dyadic quality of the interplay. Furthermore, in line with previous literature that depicted the Non-Hostility scale as particularly stable and difficult to detect compared to the other scales [90,102] we did not find changes over time. In fact, an expert observer may also have some difficulties in noting subtle and covert signs of hostility (e.g., boredom, discomfort) in an adult’s tone of voice or in face, and more time for an appropriate and accurate evaluation is needed [102]. Also, even if parents are particularly uncomfortable during the interaction, they are aware of the fact that they are video-recorded, and for this they unlikely will show clear signs of hostility and/or aggression toward the child. However, our results highlighted the substantial absence of hostile behaviors and signs, with scores being in the range “moderate–good” both pre- and post-intervention [38,102].

The child’s increases in the responsiveness domains might be influenced by parental involvement, which gives them the possibility to experience positive exchanges, first of all mediated by the therapist and then gradually alone with the child. In this sense, the therapist somehow acts as a promoter of the exchange, supporting both the partners in establishing effective and adaptive social routines in which they can experiment themselves in a functional and pleasant manner. Therefore, the therapist gradually leaves space to the dyad, supporting them only when necessary to facilitate reciprocal attunement. Experimenting with these positive changes, parents might be more motivated and facilitated to reproduce specific social routines also in the domestic context.

Concerning the child’s scales, in general we expected that a specific work on adult abilities would impact also on child’s abilities to respond positively to the more adequate and functional proposals. On the basis of our hypothesis and considering the dyadic nature of the relationship in which both the partners play an active role, we also expected a subsequent increase in the child’s Responsiveness scale. In fact, the significant increases found in parents may indicate having acquired better abilities to understand, interpret and respond to their communicative signals, often impaired or atypical in ASD.

In line with this, results in literature showed how children exhibit better play skills when the activities were scaffolded by an adult [44,103]. Within a more supported environment, children are provided more opportunities to request and comment on objects than they would have if they played alone. Coherently, our results revealed an increase in child’s general affective regulation, even if scores are still in the inconsistent range of the Responsiveness scale. In fact, it may be still present a quality of inappropriateness and the child may get dysregulated in front of particularly challenging situations. Nevertheless, the general amount of the child’s responsiveness to parents’ initiative significantly increased over time, and this was also confirmed by the significant increase in the subscale that assesses the amount of physical contact sought by the child. The framework of the intervention focuses on two main aspects: establishing virtuous circles in the dyad and promoting intentionality and reciprocity. The results discussed so far seem to support the first one. With respect to the second, results seemed to depict a more intentional child. In fact, even if general levels of the Involvement scale generally fall into the “inconsistent–low” range both before and after intervention, it is interesting to notice that children significantly enhanced their involving strategies towards their caregivers. Child’s involving strategies comprise: the use of non-verbal channels such as eye-contact or verbal involvement, using both talking and babbling, and also through the physical positioning of their body in a way that does not exclude the other. These results suggest an enhancement in child’s functional strategies to involve the social partner, effectively expressing communicative intentionality.

As we expected, the child responds to more effective caregiver strategies progressively increasing his/her degree of motivation to begin socio-communicative routines in the first place, therefore actively engaging the social partner in the interchange.

As highlighted by literature, more functional and effective interactions also have an impact on cognitive abilities and, in general, on child development [2,3] also in the context of ASD [8,14,16]. Further, it is well established that play skills are related to cognitive skills and play is considered as a primary opportunity of learning, especially when shared in the context of child–adult interactions [102]. On this basis, in the context of intervention, shared play represents a key mediator of the whole process [10]. In line with this, children did not show significant changes in the quantity of play, demonstrated by the fact that “non-play levels” seem to be stable before and after the intervention. Nonetheless, as we expected, child’s play exhibited significant changes in quality over time. In fact, we found out a significant increase in children’s symbolic play together with the reduction in the levels of the exploratory play. Hence, results suggest a significant evolution in the quality of play in the direction of the typical milestones of play development. Interestingly, mothers’ and children’s play levels are related to each other. In fact, child’s exploratory play was moderately correlated with that of the mother after intervention, but not before, suggesting the presence of a greater ability of dyadic synchronization. Further, mothers’ and children’s symbolic play levels resulted to be significantly correlated both before and after intervention. Even more importantly, correlation increases from moderate to strong over time with respect to symbolic play. This change goes in the direction of the reinforcement of higher levels of reciprocal syntonization, supporting the importance of directly intervening on the dyad to promote bidirectional exchange.

So far, we pointed out significant changes in affective and cognitive aspects of the relationship in the context of intervention. In line with previous literature, we investigated both child and caregivers’ factors that might be relevant for the intervention. Particularly, we expected that parental stress levels could have an influence on adult Emotional Availability. We found out that the general stress levels seemed not predictive of parents’ abilities. Interestingly, among the different subdimensions of parental stress, a significant negative relationship emerged between the perception of having a difficult child and the ability of the caregiver to adequately structure the interaction. From a clinical standpoint, this result might emphasize the relevance of caregivers’ perception of the child, as well as the need of taking into account parents’ mental representation during the implementation of the parental based early intensive intervention.

Further, we also explored the impact of child’s variables on caregivers’ behaviors. From our analysis, it emerged that the child’s language communicative abilities seemed to be positively associated with parents’ ability to not be intrusive, interfering with the child’s activity. In addition to this, linguistic and communicative elements seemed to impact also on the child’s relational aspects, in particular in his/her modalities to respond to the mother. More specifically, language abilities seemed to be in turn linked to play abilities. In particular, greater symbolic play skills appear to be predictive of better language. From this analysis, it emerges that linguistic aspects showed a general influence on both parent and child interactive behaviors. In particular, when a child has the possibility to directly communicate in a more effective way, the parent, in turn, gives the impression to show a lower necessity to directly interfere with his/her activity. It is also worth noting that, in line with literature on developmental psychology, our results seemed to support the role of play competencies in cognitive development, which in turn entirely happens in the context of the interpersonal relationship mediated by affective exchanges.

### Limitations

This study presents some limitations. A main limit of the present work is represented by the small sample size, consequently, further studies should be conducted with larger samples size in order to achieve more generalizable results. Our study also focused only on mothers, while fathers may show different characteristics and changes. Moreover, our sample is unbalanced with respect to gender and therefore, this limits the generalization of the results to females, that may have different characteristics. Another main limitation consists in the absence of a comparison group subjected to an intervention with a different theoretical framework (e.g., treatment “as-usual”) or another clinical group that was not exposed to any intervention. However, given the importance of guaranteeing an early intervention as soon as possible especially for preschool children with neurodevelopmental disorders, waiting lists were not a possibility. Another limitation of this study concerns the presence of only two time points for the evaluation of the dyadic changes, in fact more assessments will be able to better describe the process of change in both affective quality and play during interventions.

In this sense, we aim to conduct next studies including a bigger sample comprising fathers and more balanced with respect to gender. We will also work on building a comparison group made by children that are regularly monitored by ODFLab, but that receive the intervention in their local community services since they come from other regions. Since such interventions usually do not provide parental involvement, this can be considered as a treatment “as usual” and therefore constitutes a suitable comparison group. Further research should also include more longitudinal time points in order to better understand trajectories’ trend. Finally, next works will include different subgroups of patients with different characteristics in cognitive functioning and symptoms severity, as well as intervention outcomes variables.

## 5. Conclusions

Investigating the longitudinal changes in parent–child affective quality and play skills during a parental based early intervention may have important implications. From a theoretical perspective, it enhances knowledge about child’s and caregiver’s variables that impact on dyadic outcomes. In particular, our findings are in line with a reciprocal interdependence between the social partners, in which play skills are linked to child’s language development that, in turn, have an impact on parent’s interactive skills. Further, these abilities seem also to be influenced by the caregiver’s perceptions and representations of their children. From a clinical standpoint, our results suggest that parental-based intervention supports and facilitates improvements in both children’s and caregivers’ affective quality and cognitive abilities. In particular, parents and children exhibit greater levels of syntonization during play, as well as a trajectory that follows the milestones of typical development towards progressively more evolved interactive modalities. Further, studying dyadic aspects allows to identify important target areas to be addressed during intervention. Through this, it may be possible to identify peculiar outcome trajectories associated with different profiles and this, in turn, might help planning individualized intervention programs that directly influence parent–child interaction. To conclude, our results support the importance of actively involving caregivers during the intervention.

## Figures and Tables

**Table 1:** Demographic Statistics of the sample.

	Mean (SD)	
	T1	T2
Child Chronological Age (months)	37.793 (9.108) range (22–55)	55.500 (13.063) range (32–81)
Child Mental Age (months)	26.750 (7.006) range (15–43)	40.760 (11.296) range (23–63)
Parental Age (years)	38.071 (4.799) range (27–45)	39.996 (5.071) range (27–46)
Socio Economic Status	35.214 (13.600) range (14.5–59.5)	35.600 (14.361) range (14.5–66.0)

**Table 2:** Developmental child’s outcomes before and after intervention.

	T1 M(SD)	T2 M(SD)	*p*-Value-*r*^2^	BF
Ados Social Affect	12.931 (2.939)	11.357 (2.599)	T (27) = 3.827, *p* = 0.0007; *r*^2^ = 0.352	BF = 46.309
ADOS Restrictive and Repetitive Behabiors	3.552 (1.824)	3.500 (1.876)	T (27) = 0; *p*-value = 1; *r*^2^ = 0	BF = 0.200
Ados Total Score	16.700 (3.809)	14.893 (3.370)	T (27) = 2.718; *p* = 0.011; *r*^2^ = 0.215	BF = 4.146
ADOS Comparison Score	6.407 (1.394)	5.786 (1.101)	T (25) = 2.476; *p* = 0.020; *r*^2^ = 0.197	BF = 2.606
GMDS-General Quotient	71.964(13.675)	75.037(21.636)	T (26) = −1.311, *p* = 0.201, *r*^2^ = 0.062	BF = 0.439
GMDS–Language and Communication Scale	54 (23.764)	69.84 (37.121)	W = 77.5, *p* = 0.023; *r*^2^ = 0.458	BF = 6.671

**Table 3:** Scales and subscales of the Emotional Availability Scales.

Adult Sensitivity	Adult Structuring	Adult Non-Intrusiveness	Adult Non-Hostility
Affect	Provides appropriate guidance and suggestions	Follows child’s lead	Adult lacks negativity in face or voice
Clarity of perceptions and appropriate responsiveness	Success of attempts	Non-interruptive ports of entry into interaction	Lack of mocking, ridiculing, or other disrespectful statement and/or behavior and general demeanor
Awareness of timing	Amount of structure	Commands, directives	Lack of threats of separation
Flexibility, variety, and creativity in modes of play or interaction	Limit setting proactively	Adult talking	Does not lose cool during low and high challenge/stress times
Acceptance	Remaining firm in the face of pressure	Didactic teaching	Frightening behavior/tendencies
Amount of interaction	Verbal vs. non-verbal structuring	Physical vs. verbal interferences	Silence
Conflicts situations	Peer vs. adult role	The adult is made to “feel” or “seem” intrusive	Themes or plat themes hostile
**Child Responsiveness**	**Child Involvement**		
Affect/emotion regulation, organization of behavior	Simple initiative		
Responsiveness	Elaborative initiative		
Age-appropriate autonomy seeking and exploration	Use of adult		
Positive physical positioning	Lack of over-involvement		
Lack of role reversal/over-responsiveness	Eye contact, looking		
Lack of avoidance	Body positioning		
Task oriented/concentrate	Verbal involvement		

**Table 4:** Descriptive and Inferential Statistics of Adult Emotional Availability Scales-Main Scales and Subscales.

	Mean (SD)		*p*-Value–R^2^	Bayes Factor
	T1	T2	T1–T2	T1–T2
Total Sensitivity	4.552 (0.588)	4.914 (0.669)	*p* = 0.002 ** r^2^ = 0.596	BF = 25.097
Sensitivity 3: Awareness of timing	1.966 (0.421)	2.207 (0.412)	*p* = 0.0262 * r^2^ = 0.454	BF = 2.952
Sensitivity 6: Amount of interaction	1.0690 (0.604)	2.138 (0.516)	*p* < 0.001 *** r^2^ = 0.669	BF > 100
Total Structuring	4.276 (0.560)	5.121 (0.529)	*p* < 0.0001 *** r^2^ = 0.892	BF > 100
Structuring 1: Appropriate guidance	4.103 (0.724)	5.034 (0.731)	*p* < 0.0001 *** r^2^ = 0.795	BF > 100
Structuring 2: Success of attempts	4.103 (0.724)	4.552 (0.985)	*p* = 0.003 ** r^2^ = 0.559	BF = 23.42
Structuring 3: Amount of structure	1.690 (0.541)	2.138 (0.516)	*p* < 0.001 *** r^2^ = 0.641	BF > 100
Structuring 5: Remaining firm during pressure	2.310 (0.471)	2.552 (0.506)	*p* = 0.023 * r^2^ = 0.433	BF = 2.952
Total Non–Intrusiveness	4.466 (0.654)	5.345 (0.769)	*p* < 0.0001 *** r_2_ = 0.886	BF > 100
Non–Intrusiveness 1: Follows child’s lead	4.379 (0.775)	4.931 (0.961)	*p* = 0.019 * r^2^ = 0.504	BF = 5.069
Non-Intrusiveness 2: Non-interruptive entry in interaction	4.552 (0.783)	5.103 (1.113)	*p* = 0.014 * r^2^ = 0.549	BF = 7.699
Non-Intrusiveness 6: Physical vs. verbal interferences	2.103 (0.409)	2.310 (0.471)	*p* = 0.019 * r^2^ = 0.454	BF = 4.031
Non-Intrusiveness 7: The adult is made to feel intrusive	2.172 (0.602)	2.552 (0.506)	*p* = 0.003 ** r^2^ = 0.586	BF = 30.755
Total Non–Hostility	5.431 (0.578)	5.379 (0.883)	*p* = ns	BF = 0.210

* *p* < 0.05; ** *p* < 0.01; *** *p* < 0.001.

**Table 5:** Descriptive and Inferential Statistics of the Child Emotional Availability Scales–Main Scales and Subscales.

	Mean (SD)		*p*-Value–R^2^	Bayes Factor
	T1	T2	T1–T2	T1–T2
Total Responsiveness	3.552 (0.817)	4.034 (0.844)	*p* < 0.001 ** r^2^ = 0.716	BF > 100
Responsiveness 1: Emotional regulation of affect and behavior	3.241 (0.872)	3.552 (1.088)	*p* = 0.022 * r^2^ = 0.432	BF = 2.880
Responsiveness 2: quantity of responsiveness	3.069 (1.163)	3.655 (1.143)	*p* = 0.001 ** r^2^ = 0.675	BF = 21.431
Responsiveness 4: Positive physical positioning	1.724 (0.649)	2.000 (0.535)	*p* = 0.036 *r*^2^ = 0.479	BF = 1.836
Responsiveness 7: Task orientation-concentration	1.448 (0.686)	1.828 (0.539)	*p* = 0.003 ** r^2^ = 0.566	BF = 30.755
Total Involvement	3.138 (0.823)	3.379 (0.690)	*p* = 0.02 * r^2^ = 0.613	BF = 24.648
Involvement 2: Elaborative initiative	1.655 (0.974)	1.828 (1.071)	*p* = 0.036 * r^2^ = 0.425	BF = 2.313
Involvement 3: Use of adult	1.448 (0.632)	1.690 (0.660)	*p* = 0.010 * r^2^ = 0.491	BF = 7.206
Involvement 5: Eye–contact looking	1.310 (0.604)	1.586 (0.733)	*p* = 0.024 * r^2^ = 0.435	BF = 2.776
Involvement 6: Body positioning	1.759 (0.689)	2.069 (0.593)	*p* = 0.009 ** r^2^ = 0.471	BF = 4.698
Involvement 7: Verbal involvement	1.517 (0.688)	2.069 (0.651)	*p* < 0.0001 *** r^2^ = 0.742	BF > 100

* *p* < 0.05; ** *p* < 0.01; *** *p* < 0.001.

**Table 6:** Descriptive and Inferential Statistics for Play skills.

	Mean (SD)		*p*-Value-R2	Bayes Factor
	T1	T2	T1–T2	T1–T2
Child Exploratory Play	0.194 (0.0715)	0.133 (0.095)	*p* = 0.007 ** r^2^ = 0.234	BF = 6.319
Child Symbolic Play	0.0721 (0.0833)	0.125 (0.115)	*p* = 0.014 * r^2^ = 0.197	BF = 3.449
Adult Exploratory Play	0.1666 (0.090)	0.0126 (0.087)	*p* = ns	BF = 0.837
Adult Symbolic Play	0.160 (0.102)	0.148 (0.124)	*p* = ns	BF = 0.231

* *p* < 0.05; ** *p* < 0.01.

**Table 7:** Results of Mixed Linear Models.

	Beta	T	*P*-Value (T)	Wald F	*P*-Value (W)	Chi ^2^ (Baseline Model)	Marginal R^2^	BIC
Model 1: Structuring						X^2^ (6) = 9.364, *p* = 0.009	0.485	55.365
Intercept	1.900	t(25,474) = 2.340	*p =* 0.027	F (27.315) = 5.344	*p* = 0.029			
Predictor 1: time	0.718	t(12.196) = 9.954	*p* < 0.0001	F (14.543) = 96.451	*p* < 0.0001			
Predictor 2: Psi–Difficult Child	0.016	t(24.079) = 2.255	*p =* 0.034	F (26.096) = 4.531	*p* = 0.043			
Age caregiver	0.050	t(21.529) = 2.510	*p =* 0 0.020	F (23.798) = 6.226	*p* = 0.020			
Model 2: Non Intrusiveness						X^2^ (5) = 26.709, *p* < 0.0001	0.400	101.400
Intercept	4.090	t(46.979) = 21.277	*p* < 0.0001	F (47.394) = 429.756	*p* < 0.0001			
Predictor 1: Time	0.771	t(26.111) = 7.045	*p* < 0.0001	F (28.052) = 48.834	*p* < 0.0001			
Predictor 2: GMDS–Language and Communication	0.008	t(50.000) = 2.677	*p* = 0.010	F (50.000) = 6.610	*p* = 0.013			
Model 3: language and communications scale						X^2^(5) = 16.218, *p* = 0.0003	0.164	508.120
Intercept	47.218	t(41.660) = 8.046	*p* < 0.0001	F (41.157) = 63.895,	*p* < 0.0001			
Predictor 1: Time	8.186	t(29.848) = 1.526	*p* = 0.138					
Predictor 2: Child symbolic play	104.376	t(46.240) = 2.810	*p* = 0.008	F (45.987) = 7.321,	*p* = 0.010			
Model 4: child Responsiveness						X^2^ (5) = 10.086, *p* = 0.001	0.260	118.020
Intercept	2.947	t(46.702) = 13.129	*p* < 0.0001	F (47.260) = 163.592	*p* < 0.0001			
Predictor 1: Time	3.22	t(25.642) = 2.504	*p* = 0.019	F (28.093) = 28.093	*p* = 0.019			
Predictor 3: GMDS-Language and communication scale	0.011	t(49.983) = 3.389	*p* = 0.001	F (49.986) = 10.590	*p* = 0.002			
